# Application of modified subtotal resection of adenomyosis combined with LNG-IUS and GnRH-a sequential therapy in severe adenomyosis: A case series

**DOI:** 10.3389/fsurg.2022.914725

**Published:** 2022-08-18

**Authors:** Zhenyue Qin, Zhiyong Dong, Huimin Tang, Shoufeng Zhang, Huihui Wang, Mingyue Bao, Weiwei Wei, Ruxia Shi, Jiming Chen, Bairong Xia

**Affiliations:** ^1^ Department of Obstetrics and Gynecology, Shaoxing Maternity and Child Health Care Hospital, Shaoxing, China; ^2^ Department of Gynecology, The Affiliated Changzhou No. 2 People's Hospital of Nanjing Medical University, Changzhou, China; ^3^ Department of Gynecology, The First Affiliated Hospital of USTC, Division of Life Sciences and Medicine, University of Science and Technology of China, Hefei, China

**Keywords:** severe adenomyosis, subtotal resection of adenomyosis, levonorgestrel intrauterine delivery system, gonadotropin-releasing hormone agonist, dysmenorrhea

## Abstract

**Background and Objective:**

Adenomyosis focus resection has always been the main surgical method for patients with uterine preservation, but its curative effect and surgical method are still controversial. We improved this method on the basis of the “double-flap method” and combined it with the levonorgestrel intrauterine delivery system (LNG-IUS) and gonadotropin-releasing hormone agonist (GnRH-a) sequential treatment to determine the clinical effect and feasibility of this scheme in the treatment of severe adenomyosis.

**Methods:**

This is a retrospective review. A total of 64 patients with severe adenomyosis were treated in the Department of Gynecology of Changzhou Second People's Hospital, which is affiliated to Nanjing Medical University, from December 2017 to September 2021. The transabdominal approach and laparoscopic approach were adopted for the purposes of treatment in this study. Hence, the patients were subdivided into the transabdominal approach subgroup and the laparoscopic approach subgroup. The hemoglobin, visual analog score (VAS) score, menstruation score, and other indices of each patient before and after treatment were observed, recorded, and analyzed.

**Results:**

All 64 patients underwent the operation successfully. After the completion of sequential treatment, the CA125 decreased significantly 1 month after the operation, the average uterine volume significantly reduced, the hemoglobin value increased to a certain extent 3 months after the operation, and the menstrual score and dysmenorrhea during the first menstruation were significantly lower than they were before the operation. After the treatment, the therapeutic results of the transabdominal approach subgroup and endoscopic approach subgroup were compared on the basis of the observed indices, and no significant difference was observed (*P* > 0.05). Only one patient had a downward movement of the LNG-IUS, and the vaginal ultrasound showed that the upper end of the LNG-IUS was approximately 1.5 cm from the bottom of the uterine cavity. The average follow-up period was 24.02 ± 11.77 months, and no lesion progression was found in any patients.

**Conclusion:**

For patients suffering from severe adenomyosis who have no pregnancy plans and require uterine preservation, transabdominal or laparoscopic subtotal resection of the focus of adenomyosis, combined with the LNG-IUS + GnRH-a sequential treatment, may be a safe and effective alternative when conservative treatments such as drugs fail.

## Introduction

Adenomyosis (AM) is a disease caused by the invasion of the endometrium into the myometrium. It is an estrogen-dependent disease (1). The clinical symptoms are progressive secondary dysmenorrhea, increased menstruation, and enlarged uterine volume, and some patients may also suffer from infertility (2). It is reported that the prevalence rate is 5%–70% (3). The radical treatment of AM is total hysterectomy, which will cause impacts on women's physical and mental conditions and life after hysterectomy to varying degrees, so it is crucial to preserve the uterus. The most commonly used operation to preserve the uterus is local focus resection. However, the high recurrence in the long term and the improvement in clinical symptoms after focal resection are worthy of attention (4–6).

Many drugs can be used to treat AM, but they cannot cure the disease, and it is easy for the disease to relapse and progress after drug withdrawal (1). Related clinical studies have confirmed that both the levonorgestrel intrauterine delivery system (LNG-IUS) and the gonadotropin-releasing hormone agonist (GnRH-a) have certain efficacy in the treatment of AM, but they both have their own adverse reactions (7–11). Because of the high recurrence and unsatisfactory clinical effect of a single treatment approach for AM, clinicians have begun to adopt combined interventions such as high-intensity focused ultrasound (HIFU) combined with the LNG-IUS (12), local focus resection with the LNG-IUS (13), and HIFU with GnRH-a (14). However, with the increase in follow-up time, the recurrence rate also increases (14, 15–17).

We found that the “double-flap method” was more effective in the operation of preserving the uterus (18–20), but this method is suitable for patients with pregnancy intention after an operation, so a part of the myometrium will still be preserved. For patients who still have a strong desire to retain the uterus after the failure of drug treatment, this study improves the surgical method to reduce the recurrence among patients after treatment. During the operation, the focus of adenomyosis is resected as much as possible, and a part of the uterine cavity is remodeled to consolidate the curative effect and reduce the recurrence by sequential treatment with the LNG-IUS and GnRH-a after the operation. The purpose of this study was to preliminarily determine the efficacy of postoperative combined drugs in the treatment of severe adenomyosis.

## Data and methods

### General data

A total of 64 patients with severe adenomyosis treated in the Department of Gynecology, the Affiliated Changzhou No. 2 People's Hospital of Nanjing Medical University, from December 2017 to September 2021 were selected. The patients suffered from severe adenomyosis as determined by auxiliary examination, and the postoperative pathological results supported the diagnosis of adenomyosis. According to the guidelines of the International Federation of Obstetrics and Gynecology, the extent of the lesion was determined. If the uterus volume affected by the lesion is <25%, it is called mild, 25%–50% is called moderate, and >50% is called severe (21).

(1) Case inclusion criteria: (1) The patient had a strong desire to preserve the uterus. (2) The conservative treatment with drugs failed. (3) The patient had no pregnancy plan. (4) The patient voluntarily accepted this procedure and signed the informed consent form.
(2) Case exclusion criteria: (1) The patient suffered from primary dysmenorrhea. (2) The patient had a malignant tumor of the reproductive system. (3) The patient was unwilling to accept GnRH-a treatment or was lost during follow-up.

### Surgical steps

**Figure 1:**
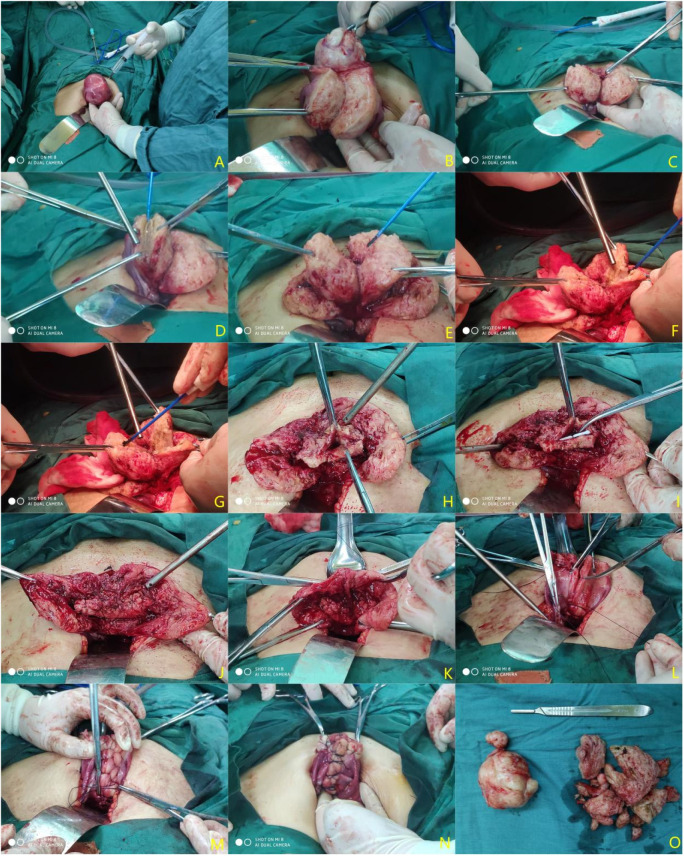
Procedure diagram of a transabdominal operation. (**A**) Inject diluted pituitrin; (**B**) remove uterine fibroids; (**C**) make a longitudinal incision of adenomyosis lesions to reach the uterine cavity; (**D**) Resect the lesion as much as possible (preserving approximately 0.5–1 cm of the plasmomuscular layer flaps); (**E**) treat the contralateral lesions with the same method; (**F**) gradually subtract the lesion to the uterine cavity and excise a part of the uterine cavity to reduce the uterine volume; (**G**) treat the contralateral lesions with the same method; (**H**) remodel the myometrium; (**I**) place the LNG-IUS (Manchester ring) and reshape the depth of the uterine cavity again based on the LNG-IUS length; (**J**) Mattress-suture the uterine cavity continuously; (**K**) align the sarcoplasmic layers and reduce the extra length; (**L,M**) suture the bilateral seromuscular layers with the “baseball stitching technique”; (**N**) repair the sutured uterus; (**O**) uterine fibroids and adenomyosis specimens resected during the operation.

Transabdominal surgery group: Make a median longitudinal incision, pull the uterus out of the pelvic cavity, wrap it with a cotton pad soaked in normal saline, and determine the location of the lesion by touching the uterus. Inject diluted pituitrin into the normal myometrium near the focal tissue (with close attention paid to blood pressure fluctuations) (Figure 1A). If the uterine leiomyoma is to be removed at the same time (Figure 1B), make a longitudinal incision with an electrotome at the location of the focus of adenomyosis to reach the uterine cavity (Figure 1C). Resect the focal lesion from the seromuscular layer separately on the myometrium flaps (Figure 1D), and retain approximately 5 mm–10 mm of the flaps for suture (Figure 1E). The lesions around the uterine cavity should be removed as clean as possible to avoid postoperative recurrence (Figures 1F,G). Finally, the endometrium and the left and right muscle layers within 5 mm were preserved as the “uterine center” (Figure 1H). Use the LNG-IUS placed during the operation as the “ruler” to reshape the uterine cavity at a depth of approximately 7–8 cm (Figure 1I). Suture the uterine cavity (Figure 1J). Align both sides of the sarcoplasmic layers and remove the extra length of these layers (Figure 1K). Suture the seromuscular layers on both sides with the “Baseball Stitching Technique” to complete the uterine plastic surgery (Figures 1L–N).

Laparoscopic surgery group: The procedure is basically the same as that of the transabdominal group. Before the operation, the location and scope of the focus were evaluated by gynecological examination, a transvaginal ultrasound, and MRI. During the operation, the focus can be resected by a rational use of energy instruments such as an ultrasonic scalpel. The specimen needs to be packed in a specimen bag and then slowly removed from the puncture hole around 1.5 cm in diameter. The “tumor-free principle” should be observed during specimen collection. Polyglactin 910: synthetic absorbable surgical suture (Model: 2-0) was used to close the uterine cavity. Polyglactin 910: synthetic absorbable surgical suture (Model: 1-0) was used to close the seromuscular layer.

After completion of either the transabdominal or laparoscopic surgery, wash the pelvic and abdominal cavity with warm saline to reduce the possibility of endometrial implantation, close the abdominal cavity layer by layer, and place drainage tubes in the pelvic cavity when necessary.

### Postoperative management

No special postoperative treatment was required for the patients. After discharge, GnRH-a injection was given on the first day of menstruation. If the menstruation did not occur due to the placement of the LNG-IUS, the first injection can be administered according to the previous menstrual cycle. Administer three to six injections according to the patient's age, disease condition, and other factors, with an interval of 28 days and regular follow-up (a total of 27 patients received 6 injections of GnRH-a, and 37 patients received 3 injections of GnRH-a).

### Observed indices

The indices before, during, and after the operation were recorded to evaluate the therapeutic effect. The indices to be observed include the following: (1) operation time and intraoperative blood loss; (2) hemoglobin value at preoperative and 3 months postoperative; (3) CA125 at preoperative and 1 month postoperative; (4) dysmenorrhea score at preoperative and the first menstrual period after GnRH-a injection; dysmenorrhea score calculated by using visual analog score (VAS) where the pain scale was subdivided into 10 grades; “no pain” was indicated on the left side of the scale, and “the maximum pain you could imagine” was designated on the right side of the scale; (5) menstrual volume score at the preoperative and the first menstrual period after GnRH-a injection, scored with the pictorial blood loss assessment chart (PBAC) (22); (6) uterine volume estimated by transvaginal ultrasound at preoperative and 3 months postoperative where the uterus size is estimated using the elliptical volume formula, which is 0.52 × length × thickness × width of the uterus measured with transvaginal ultrasound (23); (7) regular follow-up with transvaginal ultrasound to check for any progress of AM lesions and displacement of the LNG-IUS.

### Statistical analysis

The data from this study were statistically analyzed using the SPSS26.0 statistical software. The results were expressed as the mean value ± standard deviation $(\bar{x}\pm s)$, though the measured variable values were not normally distributed. Then, the *t*-test was performed on the observed indices of the 64 patients before and after the operation. If *P* < 0.05, it means the difference was statistically significant.

## Results

### Demographic details

A total of 64 patients received and completed this combined treatment, and the baseline characteristics of all these patients are given in Table 1. The average age of the 64 patients was 42.20 ± 5.39 years. The average duration of clinical symptoms such as dysmenorrhea and menorrhagia was 5.02 ± 4.72 years, and the average BMI was 24.02 ± 3.59 kg/m^2^. Among them, 36 patients had endometriosis and 18 patients had leiomyoma of the uterus, 9 patients had both, while 19 patients had only adenomyosis without uterine leiomyoma or endometriosis.

**Table 1:** Basic information on 64 patients.

Characteristics	Total (*n* = 64)
Mean age ± SD, years	42.20 ± 5.39
Average time with clinical symptoms ± SD, years	5.02 ± 4.72
Preoperative BMI ± SD, kg/m^2^	24.02 ± 3.59
Fertility history, *n* (%)	
*n* = 0	2 (3)
*n* = 1	41 (64)
*n* ≥ 2	21 (33)
Abortion history, *n* (%)	
*n* = 0	14 (22)
*n* = 1	13 (20)
*n* ≥ 2	37 (58)
Complications, *n* (%)	
Leiomyoma	18 (28)
Endometriosis	36 (56)
Leiomyoma and endometriosis	9 (14)
No leiomyoma and endometriosis	19 (30)

### Pre- and post-treatment outcomes

(1) The hemoglobin value remeasured 3 months after the operation increased to a certain extent compared with that before the operation, and the difference was statistically significant (*P* = 0.000) (see Table 2). (2) A total of 50 patients out of the 64 patients before the operation tested positive for CA125. The remeasured CA125 1 month after the operation was lower than that before the operation, and the difference was statistically significant (*P* = 0.000) (see Table 2). (3) The degree of dysmenorrhea after the completion of GnRH-a sequential treatment was lower than that before the operation, and the difference was statistically significant (*P* = 0.000) (see Table 2). (4) The menstrual volume score after completion of GnRH-a sequential treatment was lower than that before the operation, and the difference was statistically significant (*P* = 0.000) (see Table 2). (5) The estimated average uterine volume re-examined 3 months after completion of the sequential treatment was smaller than that before the operation, and the difference was statistically significant (*P* = 0.000) (see Table 2). (6) During the postoperative follow-up, only one patient developed an intrauterine device migration, and the vaginal ultrasound showed that the upper end of the birth control ring was approximately 1.5 cm away from the bottom of the uterine cavity. The average follow-up lasted 24.02 ± 11.77 months, and no disease progression was observed.

**Table 2:** Changes in observed indices pre-treatment and post-treatment of 64 patients $(\bar{x}\pm s)$.

Observed indices	Pre-treatment	Post-treatment	*P*
Hemoglobin value (g/L)	104.77 ± 19.41 (56–144)	118.08 ± 9.67 (105–142)	.000
CA125 (U/ml)	92.64 ± 104.45 (9.38–245.8)	14.18 ± 8.95 (4.58–45.36)	.000
Dysmenorrhea score (points)	8.13 ± 0.75 (7–9)	1.36 ± 0.65 (0–3)	.000
Menstrual volume score (points)	131.42 ± 13.25 (106–166)	22.52 ± 9.18 (3–48)	.000
Uterine volume (cm^3^)	173.61 ± 76.49 (76.58–340.70)	44.98 ± 16.97 (16.47–82.19)	.000

Hemoglobin value is for 3 months post-treatment; CA125 is for 1-month post-treatment; dysmenorrhea score and menstrual volume score are for the first menstrual period after GnRH-a injection; uterine volume is for 3 months post-treatment.

### Operation time and blood loss

None of the 64 patients had any complications during or after the operation. There was no significant difference in the operation time between the two subgroups (*P* > 0.05). There was a statistically significant difference in intraoperative blood loss between the two subgroups (*P* = 0.000) (see Table 3).

**Table 3:** Operation time and intraoperative blood loss of subgroups $(\bar{x}\pm s)$.

Operation-related information	Subgroups		
	Transabdominal surgery group (*n* = 31)	Laparoscopic group (*n* = 33)	*P*
Operation time (min)	143.71 ± 32.51 (75–230)	134.55 ± 46.10 (70–260)	.360
Intraoperative blood loss (ml)	198.06 ± 145.93 (30–500)	54.85 ± 43.02 (20–200)	.000

### Pre- and post-treatment outcomes of the two subgroups

After the treatment was completed, the abdominal approach treatment of this scheme was completed, and various observation indices improved after treatment (*P* = 0.000). The laparoscopic approach treatment of this scheme was completed, and the observation indices also improved after treatment (*P* = 0.000). The differences in the observed indices between the two subgroups were not statistically significant (*P* > 0.05) (see Table 4).

**Table 4:** Changes in observed indices pre-treatment and post-treatment of a transabdominal surgery group and a laparoscopic surgery group $(\bar{x}\pm s)$.

	Pre-treatment		Post-treatment		*P*	*P* *
	Transabdominal surgery group (*n* = 31)	Laparoscopic surgery group (*n* = 33)	Transabdominal surgery group (*n* = 31)	Laparoscopic surgery group (*n* = 33)		
Hemoglobin value (g/L)	99.71 ± 18.40 (72–138)	109.52 ± 19.41 (79–144)	117.13 ± 10.38 (102–142)	118.97 ± 9.01 (102–135)	.000	.000
CA125 (U/ml)	111.55 ± 133.96 (11.71–749.90)	74.87 ± 63.10 (17.38–249.80)	12.54 ± 8.53 (4.58–45.36)	15.71 ± 9.18 (7.36–45.35)	.000	.000
Dysmenorrhea score (points)	8.39 ± 0.67 (7–9)	7.68 ± 1.38 (7–9)	1.23 ± 0.67 (0–2)	1.48 ± 0.62 (1–3)	.000	.000
Menstrual volume score (points)	130.29 ± 12.94 (105–162)	129.09 ± 24.06 (106–166)	21.87 ± 7.50 (3–31)	22.76 ± 10.50 (3–48)	.000	.000
Uterine volume (cm^3^)	198.80 ± 80.64 (87.55–340.70)	147.20 ± 63.80 (76.58–335.95)	38.83 ± 15.14 (16.47–73.36)	49.51 ± 17.08 (14.71–80.70)	.000	.000

Hemoglobin value is for 3 months post-treatment; CA125 is for 1-month post-treatment; dysmenorrhea score and menstrual volume score are for the first menstrual period after GnRH-a injection; uterine volume is for 3 months post-treatment. *P*: Statistical analysis of a transabdominal surgery group pre-treatment and post-treatment.

* *P*: Statistical analysis of a laparoscopic surgery group pre-treatment and post-treatment.

## Discussion

According to the data in Table 1, the average age of our 64 patients is 42.20 ± 5.39 years. This is similar to the fact that most of the reported patients are over 40 years old, and the age of onset is getting younger (24–26). The average BMI value is 24.02 ± 3.59 kg/m^2^, which is a bit high, and it has been reported that an increase in BMI values increases the risk of uterine leiomyoma (27, 28), but whether AM is associated with patient weight gain may be an issue worth exploring. The data indicate that two patients did not have children, one of whom had two miscarriages, and the other had no history of abortion. A total of 62 (97%) patients had a history of childbearing, and 50 (78%) had a history of miscarriage, which was also related to the fact that fertility and abortion may be the causes of AM (29). In order to reduce AM incidence, we should adopt good contraceptive measures to reduce unnecessary abortions. The data indicated that a total of 18 (28%) patients were complicated with leiomyoma, which was similar to the reported 15%–57% (30), and 36 (56%) patients were complicated with endometriosis. Some studies have suggested that the incidence of adenomyosis with deep infiltrating endometriosis (DIE) is within 6.8%–25.4% (31). The reason for this may be related to our careful exploration of pelvic endometriosis, especially the occurrence of DIE.

Hysterectomy is still the main radical method for patients with severe adenomyosis. However, the uterus is a unique organ for women, especially in China where hysterectomy is not accepted by most patients, and this mindset is also observed in Japan (32). With the progress and improvement of conservative surgical methods in recent years, the double-flap method and the triple-flap method can not only preserve the uterus but also improve the short-term effects of dysmenorrhea, excessive menstruation, and infertility (19, 20). However, the boundary between the focus of AM and normal myometrium is not clear, and the focus cannot be completely removed during the operation (33). The residual small lesions may grow slowly and recur (34, 35). A 2-year follow-up study showed that the recurrence rate of patients with adenomyosis treated with GnRH-a was 28.1%, which was lower than the 49% for treatment with surgery alone (36). The LNG-IUS can release progesterone directly into the uterine cavity. In recent years, many studies have confirmed that the LNG-IUS can relieve symptoms such as dysmenorrhea and menorrhagia in patients with AM and reduce the level of CA125 to a certain extent (9, 23, 37). However, some studies have found that the slippage rate among AM patients with a larger uterus is as high as 37.5% after the implantation of the LNG-IUS (38). It can be seen that the therapeutic effect of the LNG-IUS on AM patients with a larger uterine volume is not as good as that of AM patients with a smaller uterine volume.

We improved the method of operation by cutting the uterus vertically to the uterine cavity, eradicating AM lesions as much as possible, and reducing the uterine cavity according to the depth of the LNG-IUS. The process of removing the focus is to change the adenomyosis from a larger volume to a smaller one. The postoperative combination with the GnRH-a therapy can reduce the recurrence rate of AM to a certain extent (36). The LNG-IUS has a better therapeutic effect on AM patients with a smaller uterine volume (9, 23, 37). The therapeutic effect of this regimen may be similar to that of the LNG-IUS in the treatment of small-volume adenomyosis. During the postoperative follow-up, only one patient had a downward displacement of the LNG-IUS, as observed with transvaginal ultrasound (transvaginal ultrasound showed that the upper end of the LNG-IUS was approximately 1.5 cm from the bottom of the uterine cavity). The LNG-IUS of the other patients was normal. During the follow-up, it is suggested that the incidence of LNG-IUS displacement is temporarily much lower than that suggested in other reports (39–42). We are of the opinion that the decrease in the LNG-IUS displacement may be related to the following: intraoperative remodeling of the uterine cavity to better align the LNG-IUS with the shape of the uterine cavity; and the postoperative injection of GnRH-a, which can reduce the uterine volume and uterine cavity again, reducing the probability of spondylolisthesis. However, it is necessary to increase the sample size and evaluate the change in the LNG-IUS slippage rate with time after the placement of the LNG-IUS. The effective time of the LNG-IUS is 5 years. If the patient is still menopausal after 5 years, a new LNG-IUS can be replaced until menopause to maintain the therapeutic effect.

It is difficult to remove the focus of adenomyosis completely because there is no obvious boundary between adenomyosis and normal myometrium. If there are residual small lesions during the operation, they may recur as time passes. A retrospective study showed that with the increase in follow-up time after surgery, the recurrence rate of patients with adenomyosis increases from the lowest (no recurrence) to the highest (close to 50%) (17). Some studies have also confirmed that GnRH-a or LNG-IUS treatment can reduce the recurrence rate (9, 23, 36, 37). Therefore, we designed this scheme, and it can be seen from Table 2 that the patients who completed sequential treatment after a modified subtotal resection of adenomyosis showed a significant improvement in postoperative hemoglobin, CA125, dysmenorrhea, menstrual volume, and uterine volume. The average uterine volume estimated by transvaginal ultrasound significantly reduced after treatment in both the transabdominal group and the laparoscopic group. The reduction of the volume of the uterus is the inevitable result of surgical resection of the focus of adenomyosis, but the author opines that it is very important to monitor the changes in the uterus volume after the operation. During the follow-up period, if the uterus volume increases with time, we need to be on guard against recurrence. Table 3 shows that the amount of intraoperative bleeding in the laparoscopic subgroup is lower than that in the transabdominal subgroup, which has something to do with the finer laparoscopic operation. However, there was no significant difference in operation time between the two subgroups. Table 4 shows that there is no significant difference in the therapeutic effect between the transabdominal approach subgroup and the endoscopic approach subgroup, considering that the improvement of the endoscopic technique of the operator may achieve a result similar to that of the transabdominal approach. Longer follow-up observation is still needed.

After the improvement of this operation and the sequential treatment with the combination of two drugs, the following advantages are observed: (1) The uterus of the patient can be preserved, the effect of cutting off the ascending branch of the uterine artery on the ovary can be avoided, the length of the vagina is not reduced, and the quality of female sex life can be preserved; (2) it retains the original normal structure around the cervix, reduces the probability of injuring the ureter, bladder, and other adjacent organs during the operation, and avoids the change of micturition and defecation habits caused by the change in the pelvic structure; (3) during the operation, the uterine cavity is repaired according to the length of the LNG-IUS, which is the difference between this operation and other uterine preservation operations. Remodeling the uterine cavity can directly and effectively reduce the amount of menstruation. Placing the LNG-IUS during the operation can also help with the treatment of AM to consolidate the surgical effect and reduce recurrence. A more appropriate size of the uterine cavity can reduce slippage of the LNG-IUS to a certain extent; (4) postoperative injection of GnRH-a can further atrophy the residual lesions, reduce the probability of abnormal uterine bleeding after the placement of the LNG-IUS, and also reduce slippage of the LNG-IUS by reducing the amount of menstruation (43).

After the improvement of this procedure, the following shortcomings are observed. (1) Endometriosis is possible during the operation, so we emphasize that the uterine cavity should be closed as soon as possible to reduce the exposure time and chance. Meanwhile, after uterine remodeling, the pelvic cavity and abdominal cavity should be fully washed to reduce the possibility of residual blood and residual endometrial cells. (2) Many tissues will be resected during the surgery, making it easy to leave dead spaces when the seromuscular layer is sutured with the myometrium retained around the uterine cavity, leading to possible hematoma and infection by errhysis. Continuous mattress suture of the uterine cavity was adopted during the operation and the bilateral seromuscular layers were sutured with the “baseball stitching technique.” This suture method can reduce needle bleeding while making the suture closer, so as to effectively avoid residual dead spaces.

In view of the fact that this study is a retrospective analysis, the number of cases is relatively small, and there may be bias in case selection, with a shorter follow-up period as well. We feel that more prospective studies with big sample sizes are also needed to evaluate and compare the efficacy and safety of this study with other treatment measures.

## Conclusion

For patients suffering from severe adenomyosis who have no pregnancy plans and require uterine preservation, transabdominal or laparoscopic subtotal resection of the focus of adenomyosis, combined with the LNG-IUS + GnRH-a sequential treatment, may be a safe and effective alternative when conservative treatments such as drugs fail.

## Data Availability

The original contributions presented in the study are included in the article/Supplementary Material, and further inquiries can be directed to the corresponding author/s.
